# Rapid Sublingual Delivery of Piroxicam from Electrospun Cyclodextrin Inclusion Complex Nanofibers

**DOI:** 10.1021/acsomega.2c03987

**Published:** 2022-09-19

**Authors:** Fuat Topuz

**Affiliations:** Department of Chemistry, Faculty of Science and Letters, Istanbul Technical University, Sariyer, 34467 Istanbul, Turkey

## Abstract

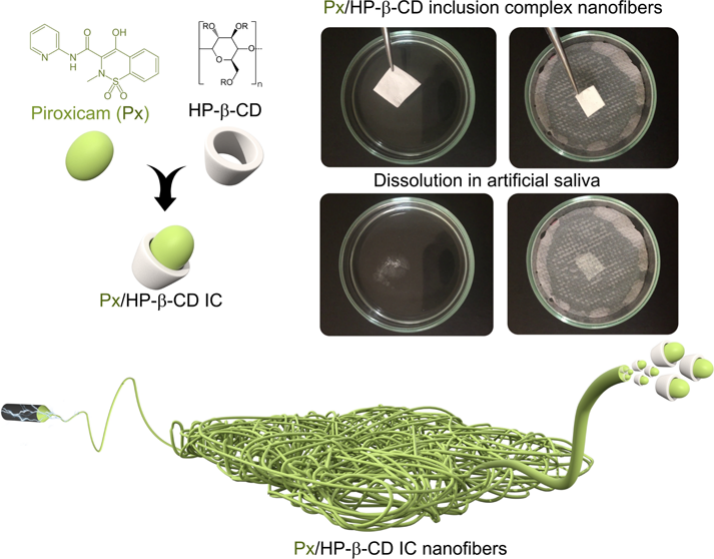

Piroxicam (Px) is a nonsteroidal anti-inflammatory drug (NSAID) used for the treatment of osteoarthritis and rheumatoid arthritis. It is administered orally; however, its poor water solubility causes low loading to the nonconventional drug delivery systems (DDSs), such as electrospun fibers. Furthermore, the rapid dissolution of DDS and fast release of the embedded drugs are crucial for oral delivery of drugs to patients who are unconscious or suffering from dysphagia. In this regard, this study reports the development of rapidly dissolving cyclodextrin (CD)-based inclusion complex (IC) nanofibers by waterborne electrospinning for fast oral delivery of Px. Scanning electron microscopy analysis revealed the formation of bead-free fibers with a mean diameter range of 170–500 nm at various concentrations of Px; increasing the Px loading decreased the fiber diameter. The formation of IC was demonstrated by X-ray diffraction (XRD) analysis by the disappearance of crystalline peaks of Px. Likewise, differential scanning calorimetry (DSC) analysis showed the disappearance of the melting peak of the embedded Px due to IC formation. Both Fourier transform infrared (FTIR) and thermogravimetric analysis (TGA) confirmed the presence of Px within the fibers. ^1^H NMR experiments demonstrated Px preservation in the fibers after six months. Px-loaded nanofibers were employed for sublingual drug delivery. To mimic the environment of the mouth, the nanofibers were treated with artificial saliva, which revealed the instant dissolution of the nanofibers. Furthermore, dissolution experiments were performed on the tissues wetted with artificial saliva, where the dissolution of the fibers could be extended to a few seconds, demonstrating the suitability of the materials for sublingual oral drug delivery. Overall, this paper, for the first time, reports the rapid oral delivery of Px from polymer-free CD fibers produced by waterborne electrospinning without the requirement of any carrier polymer and toxic solvent.

## 1 Introduction

Piroxicam (Px) is a nonsteroidal anti-inflammatory drug, which is used for reducing swelling, pain, and joint stiffness from arthritis.^1,2^ Tablets or capsules are used for oral administration of Px to relieve the symptoms of painful conditions.^3^ However, many elderly patients find it difficult to swallow tablets and capsules, especially patients suffering from dysphagia.^4^ Likewise, the delivery of drugs to unconscious patients with standard swallowing tablets becomes problematic. The need for rapid oral delivery of Px, particularly for unconscious patients and people with dysphagia, led to bringing out novel and safer drug delivery systems, which could release the embedded Px on contact with saliva. In this regard, the delivery of Px *via* the sublingual route appeared as a helpful approach when a rapid onset of the action is required. Since the sublingual area of the mouth is more permeable than the buccal (cheek) and palatal (roof of the mouth) areas, the drug molecules can be readily absorbed through the sublingual blood vessels resulting in acceptable bioavailability of the drugs.^5−7^ In this regard, the development of the oral drug delivery system without the need for any toxic solvents and reagents is essential.^8,9^ Likewise, no toxic ingredients should be involved in this process. Due to Px’s poor solubility in water, it can be dissolved using organic solvents, surfactants, or acids to boost its loading content in the drug delivery systems.^10,11^ However, this might cause adverse health effects on the oral administration of the Px drug over solvent traces. In this context, the use of pharmaceutical excipients, such as cyclodextrin (CD) for poorly soluble drugs (herein Px), can drastically boost its bioavailability without the requirement for a toxic solvent or a carrier polymer matrix.^12^

The electrospinning technique offers great potential for the development of nanofibrous oral drug carriers. In this regard, electrospun nanofibers could be produced using a single fluid,^13,14^ coaxial,^15^ triaxial,^16^ and other complex processes.^17^ Such processes lead to various structures from hollow to Janus and their combinations.^18^ In the electrospinning process, drug and polymer carriers are mixed to form a homogeneous mixture in an appropriate solvent system, just like the traditional casting method, followed by the electrospinning of solution into nanofibers.^19^ In one example, tri-section Janus nanofibers were produced through tri-fluid electrospinning and used for the delivery of water-soluble Chinese medicine, helicide.^17^ Release studies revealed that sucralose (*i*.*e*., used for sweet taste) could be released before the release of helicide takes place. Another coaxial electrospinning system was used for the rapid dissolution of dichlofenac sodium.^20^ Nanofibers with core–sheath structures, in which the drug/polymer core was encapsulated by the sheath sucralose/polymer composites. The artificial tongue experiments showed that the release process took less than 1 min.

Because of its size and hydrophobic nature, Px can form host–guest complexes with β-CD molecules.^21−23^ Even the protonated form of Px could form more effective complexes. The complexation of Px with hydroxypropyl (HP) β-CD was also reported, where the crystalline peaks of Px were hindered in the X-ray diffraction (XRD) pattern of the final formulation.^24^ Such hosting in the CD cavity enhances the stability of Px and boosts its bioavailability in physiological solutions.^24^ Since the complexation between the CD and Px boosts its solubility and stability, CD has been employed as a pharmaceutical excipient to develop many Px delivery systems. In one example, Px/β-CD inclusion complexes (ICs) could be loaded into cellulose-based microspheres for controlled release.^25^ The ICs were embedded into microparticles produced from pure Px and ethylcellulose or ethylcellulose and hydroxypropyl methylcellulose using an organic solution emulsified in water. The drug release tests were performed in an acidic medium (pH = 1.2 and 37 °C) where the samples showed a burst release, followed by a gradual release of Px. Rahmani et al. embedded Px into the polycaprolactone nanoparticles through an emulsion using a dichloromethane/water system.^26^*In vitro* release studies revealed the gradual release of Px from nanoparticles without any burst release.

Px could also be embedded in electrospun fibers for delivery. In one example, Px-loaded electrospun biodegradable nanocomposite scaffolds were produced for periodontal regeneration.^27^ Px could be encapsulated in the chitosan/poly(vinyl alcohol) (PVA)/hydroxyapatite electrospun fibers, where Px was added to the aqueous acetic acid suspension of hydroxyapatite/chitosan/PVA and then homogenized using an ultrasonicator. The solution was electrospun into fibers, and the burst release of Px was observed. Abpeikar et al. reported Px-loaded gelatin nanofibers for meniscus cartilage repair.^28^ For Px loading, first gelatin was dissolved in acetic acid solution and then mixed with the Px ampule. The nanofibers were cross-linked with glutaraldehyde and employed for coating polycaprolactone/polyurethane scaffolds, which could successfully accelerate meniscus regeneration. In another study, the fast dissolving oral film of Px by the solvent casting technique using crospovidone/sodium starch glycolate as super disintegrating agents, β-CD/Px IC, chitosan, and sodium carboxymethyl cellulose as film-forming agents were reported.^29^ Because of the hydrophilic nature of the components, the total release of Px took less than 2 min. Another fiber-based system using hydroxypropyl methylcellulose and polydextrose was developed for Px release.^30^ They observed that the recrystallization of Px in a microcrystalline form instantly after wetting could result in higher drug dissolution. All of these studies rely on using a polymer carrier to develop a drug delivery system. To the best of our knowledge, there is no study on waterborne, polymer-free electrospinning for the oral delivery of Px, which can provide rapid and safer delivery of Px in the mouth without the need for any organic solvent. In this regard, CD-based nanofibers offer promising properties since they do not require a polymer carrier or organic solvent while benefitting from high drug loading capacity due to inclusion complexation and rapid dissolution.^31^ The formation of inclusion complexation with CD molecules will boost the bioavailability of drugs,^32−34^ while masking the bitter and disgusting taste of drugs.^35−39^ Moreover, HP-β-CD received FDA approval as a vehicle for drug delivery and was incorporated into the FDA’s list of Inactive Pharmaceutical Ingredients.^40^ Thus, polymer-free HP-β-CD-based nanofibers have been used as a carrier of many drugs molecules, but not for piroxicam.^41−46^

**Figure 1:**
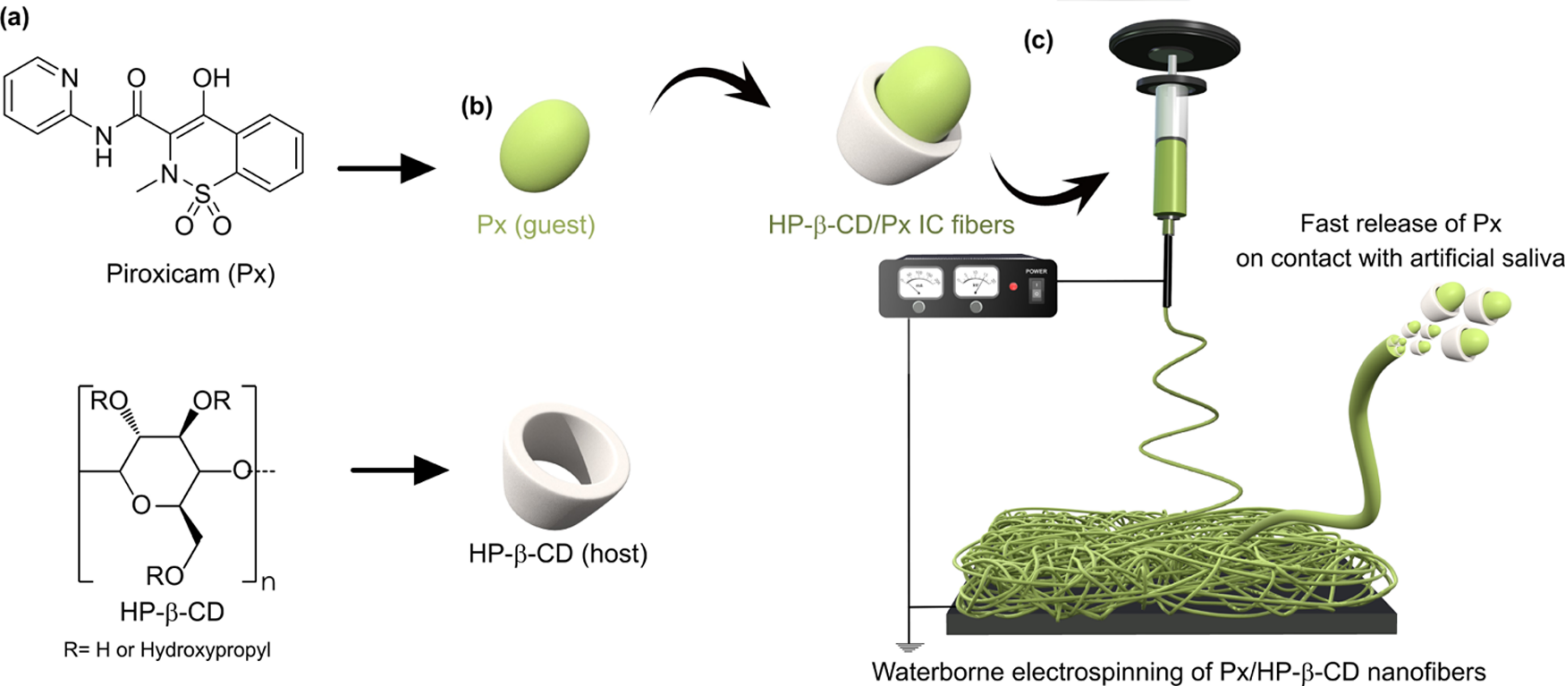
(a) Chemical structures of HP-β-CD and Px and (b) a cartoon showing the inclusion complexation between the HP-β-CD and Px. (c) The production of nanofibers by waterborne electrospinning of IC solutions to be used for the sublingual delivery of Px.

In this study, rapid sublingual delivery of Px using waterborne electrospinning of ICs of HP*-*β*-*CD and Px was reported (Figure 1). The morphology of the resultant fibers was explored through scanning electron microscopy analysis. The embedment of Px within the fibers was tested through Fourier transform infrared (FTIR) analysis and thermogravimetric analysis (TGA). The formation of the inclusion complexation between the CD and Px was explored through XRD and DSC. The preservation of Px within the fibers after six months was investigated through NMR analysis. The dissolution of the Px-loaded fibers was tested in artificial saliva and on wetted tissues to mimic the sublingual delivery of drugs.

## 2 Experimental Section

### 2.1 Materials

HP-β-CD (pharmaceutical grade) was obtained from Ashland Chemicals. Piroxicam (Px) (USP, 97–103%, Spectrum Chemical) was purchased and used as received. Sodium chloride (NaCl, ≥99.0%, Merck), sodium phosphate dibasic heptahydrate (Na_2_HPO_4_, ≥99.0%, Sigma Aldrich), potassium phosphate monobasic (KH_2_PO_4_, ≥98.0%, Sigma Aldrich), and o-phosphoric acid (75% Emprove Expert, Sigma Aldrich) were obtained commercially. The aqueous solutions of HP-β-CD and Px were prepared using Milli-Q Type II water.

### 2.2 Production of Polymer-free Cyclodextrin Inclusion Fibers

First, the inclusion complexes of HP-β-CD and Px were prepared by mixing for 1 day in water. After that, the solutions were transferred to the syringe’s joint with a blunt needle (20G). The syringes were placed on a syringe pump, and the feeding rate was 0.5 mL h^–1^, while the tip-to-collector distance was set to 15 cm. The applied voltage was set to 15 kV. During the electrospinning process, the relative humidity was 50–54%, and the temperature was 24 °C. Once the electrospinning was completed, the electrospun mats were characterized by several techniques.

### 2.3 Characterization

The morphology of the Px powder and the electrospun nanofibers was explored using a scanning electron microscope (Zeiss LEO Supra 35VP) at 5 kV. The samples were sputtered with a thin layer of Pd/Au before SEM analysis. The Fourier transform infrared (FTIR) spectra of the samples were recorded on a Thermo Nicolet 6700 spectrometer equipped with an ATR sampling accessory. The spectra were recorded for 128-scan accumulation for an acceptable signal/noise ratio at a resolution of 4 cm^–1^. Thermal analysis of the materials was carried out using a Shimadzu Corp. DTG-60H (TGA/DTA) by heating the samples to 600 °C at a rate of 10 °C/min under a nitrogen atmosphere. Differential scanning calorimetry analyses of the samples were performed on a DSC 2000 (TA Instruments) through a heating–cooling cycle up to 270 °C with a heating/cooling ramp rate of 10 °C min^–1^. The data were analyzed using Trios software (TA Instruments). Wide-angle X-ray diffraction analysis of the samples was performed on a RIGAKU Smartlab diffractometer in the 2θ range of 4–40°. The data were analyzed using high X′Pert HighScore analysis software (version 2.0a). ^1^H NMR and ^13^C NMR analysis of the samples was performed on an Agilent VNMRS 500 MHz nuclear magnetic resonance spectrometer. The samples were dissolved in D_2_O. Each spectrum consisted of 128 scans for ^1^H and 8000 scans for ^13^C analysis.

### 2.4 Dissolution of Electrospun Fibers

For the dissolution tests, artificial saliva was prepared as reported.^41^ In brief, the artificial saliva was produced using Na_2_HPO_4_ (1.19 g), NaCl (4 g), and KH_2_PO_4_ (0.095 g) in Milli-Q Type II water (500 mL). The pH of the respective solution was tuned to 6.8 with the addition of phosphoric acid. The mats were either immersed in artificial saliva directly or placed on tissues wetted with artificial saliva. The dissolution process was followed using a digital camera, and the images were cut from the respective videos to show the dissolution process (Supporting Information Videos 1 and 2).

### 2.5 Drug Release Studies

The time-dependent drug release from the nanofibers was achieved as reported before.^46^ Briefly, the fiber samples (5 mg) were placed in a PBS solution (pH: 7.4) and shaken in an incubator at 150 rpm at 37 °C. Then, 200 μL aliquots were taken from the solutions at set time intervals and replaced by the fresh PBS buffer. The measurements were carried out using a UV–vis spectrophotometer (C-7000V, Peak Instruments) in the wavelength range of 200–600 nm. The peak intensity at 360 nm was used to calculate the released Px percentage with time. The released Px percentage was calculated from the calibration curve of Px (*R*^2^ ≥ 0.99). The experiments were performed in triplicate.

## 3 Results and Discussion

**Figure 2:**
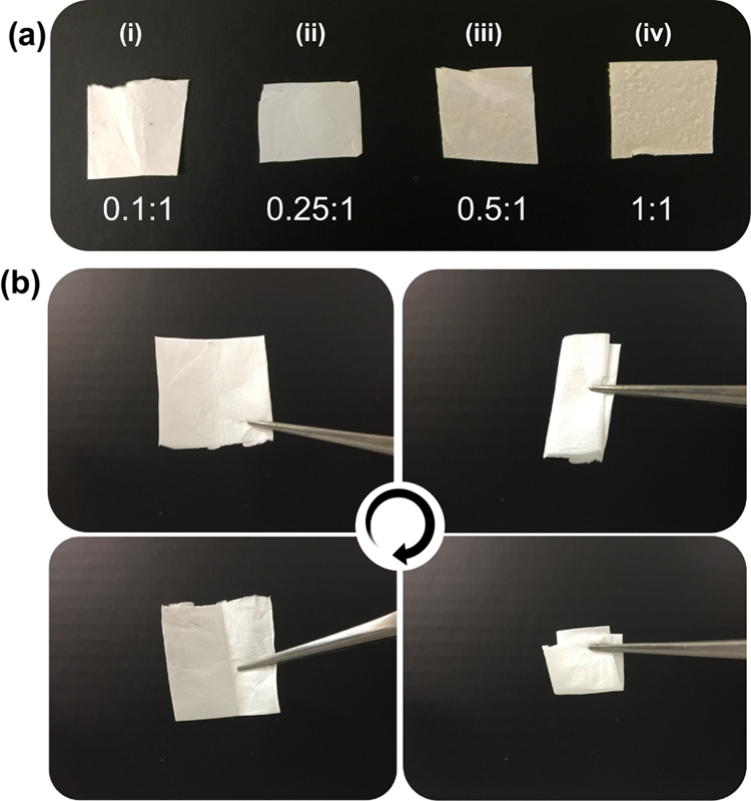
(a) Optical photos of the HP-β-CD/Px mats prepared at various Px:HP-β-CD molar ratios ((i) 0.1:1; (ii) 0.25:1, (iii) 0.5:1, (iv) 1:1). (b) Photos of the Px:HP-β-CD mat (0.25:1) during folding many times.

The inclusion complexes of the HP-β-CD and Px were prepared in water by mixing for one day at various Px:HP-β-CD (0.1–1:1) molar ratios, where homogeneous solutions without any precipitation were observed as a result of inclusion complexation between Px and HP-β-CD (Figure S1). The electrospinning of the solutions led to electrospun mats (Figure 2). The color of the mats was related to the loading concentration of Px; the electrospun mat prepared with the highest concentration of Px was pale yellow because of Px color, while at the lowest concentration, the color of the mat was white (Figure 2a). Despite the polymer-free nature, the electrospun mats of HP-β-CD/Px could be folded and twisted without rupture development (Figure 2b). This might be attributed to the presence of several hydrogen bonds between the CD molecules, which could keep the CD molecules together to maintain the fiber structure.

**Figure 3:**
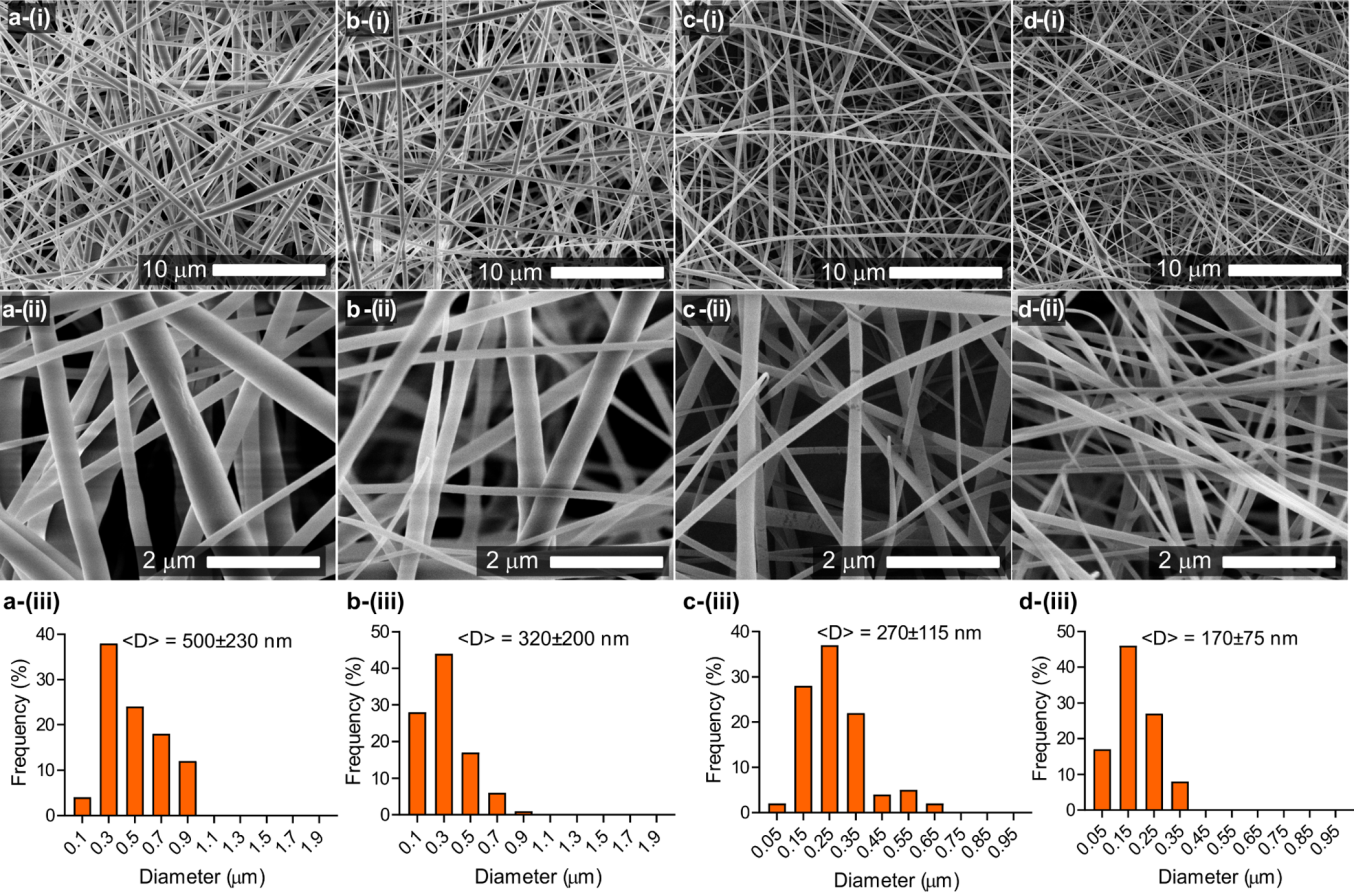
Scanning electron microscopy images of HP-β-CD/Px nanofibers prepared at various Px:HP-β-CD molar ratios: (a) 0.1:1, (b) 0.25:1, (c) 0.5:1, and (d) 1:1. The bottom panel shows the size-distribution plots of the respective nanofibers.

The morphology of nanofibers and Px powder was explored through scanning electron microscopy (SEM) analysis. The SEM image of Px powder shows the presence of crystals with rounded edges in diverse size ranges above microns (Figure S2). This is in line with the previous reports, where the Px powder has a similar structure.^47,48^ The waterborne electrospinning of HP-β-CD at 180% (w/v) led to beaded fibers (Figure S3). On the other hand, the incorporation of Px led to bead-free fibers (Figure 3). Unlike HP-β-CD fibers, thinner fibers were formed with the addition of Px. Further increasing the Px content caused a decrease in the fiber diameter; the mean diameters of the HP-β-CD prepared at various Px:HP-β-CD molar ratios (*i*.*e*., 1:0.1, 1:0.25, 1:0.5, and 1:1) were respectively calculated to be 500 ± 230, 320 ± 200, 270 ± 115, and 170 ± 75 nm. This might be attributed to the increase in conductivity of respective solutions, which led to higher stretching during jet formation, eventually ending up with thinner fibers on the collector. Similar findings were observed for the electrospun fibers of the cuminaldehyde/HP-β-CD inclusion complex, where the incorporation of cuminaldehyde led to higher stretching because of higher conductivity.^49^ High magnification SEM images revealed the smooth fiber texture without wrinkles on the fiber surfaces. This might be due to the steady evaporation of water molecules during the jetting process.

**Figure 4:**
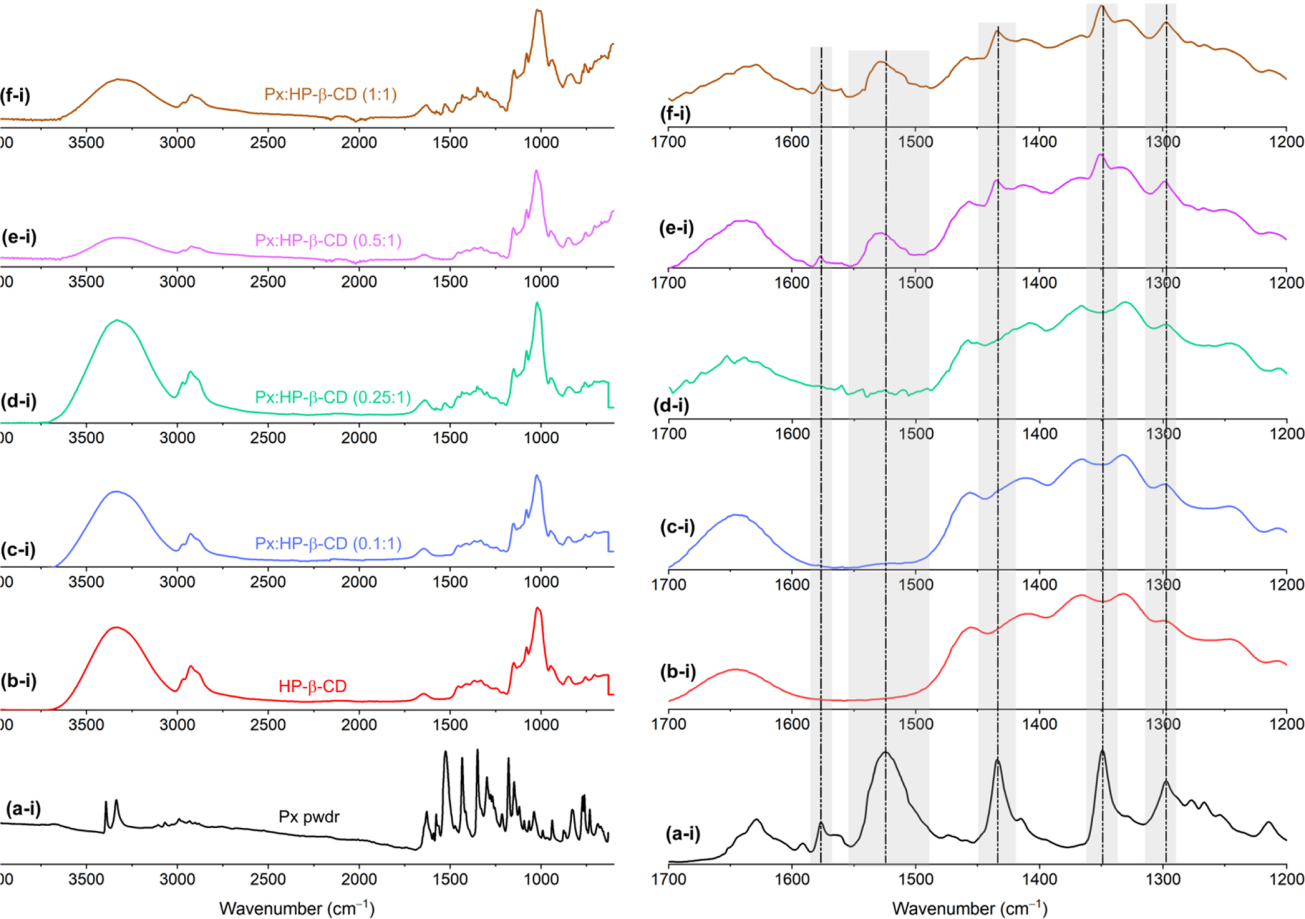
(Left panel) FTIR spectra of the (a) Px powder, (b) HP-β-CD fibers, and (c–f) Px/HP-β-CD fibers ((c) 0.1:1, (d) 0.25:1, (e) 0.5:1, and (f) 1:1), and (right panel) displays the narrow range of the respective FTIR spectra of the materials.

The embedment of Px was confirmed by FTIR analysis. Figure 4 shows the FTIR spectra of Px powder, HP-β-CD, and HP-β-CD/Px fibers. The peaks at 3341 and 2970–2930 cm^–1^ can be attributed to the OH and C–H vibration bands of HP-β-CD, respectively. While the peaks between 1200 and 1000 cm^–1^ are due to C–O–C, C–C–O, C–O, and C–C–C asymmetric vibration bands of HP-β-CD, respectively.^50^ On the other hand, the SO_2_ group of piroxicam gave bands at 1348 and 1180 cm^–1^ due to symmetric and asymmetric vibrations, while benzene (ν(C=C)) vibrations appeared as peaks at 1576 and 1434 cm^–1^. The deformation vibration of the NH group gave a band at 1525 cm^–1^. These characteristic bands of Px in the HP-β-CD/Px fiber spectrum confirm the successful embedment of Px within the fibers (Figure 4). Furthermore, the slight shifts in the peak maxima positions demonstrate the presence of interactions between HP-β-CD and Px.

**Figure 5:**
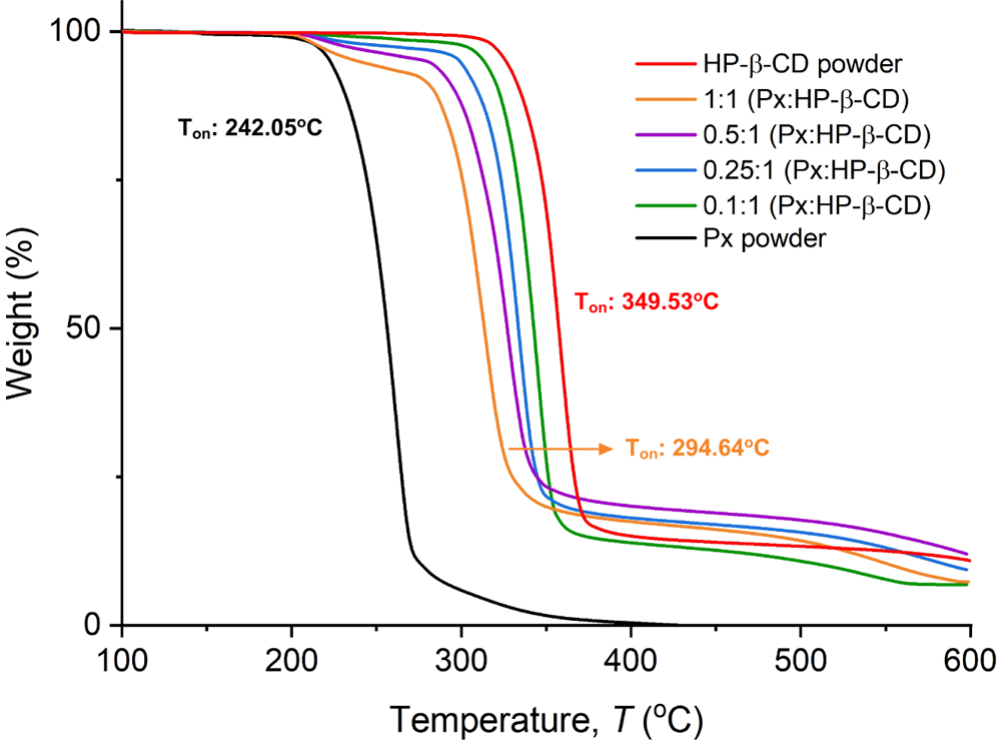
Normalized TGA thermograms of the Px powder, HP-β-CD, and HP-β-CD/Px nanofibers of various molar ratios indicated.

The embedment of Px was also confirmed by thermogravimetric analysis by the decomposition samples through heating to 600 °C at a temperature ramp rate of 10 °C min^–1^. Figure 5 shows the TGA thermograms of the Px powder, HP-β-CD, and Px/HP-β-CD fibers. HP-β-CD is a thermostable molecule and starts to decompose at ∼350 °C,^41^ while Px decomposes at around 242 °C, which is in line with the literature report.^51^ The mass loss below 300 °C can be attributed to the decomposition of the embedded Px in the fibers. The HP-β-CD/Px fibers showed a first mass loss of around 240 °C, attributed to the decomposition of embedded Px. The ratio of mass loss was proportional to the embedded Px content. Inclusion complexation boosted the thermal stability of Px, while decreasing the thermal stability of HP-β-CD seen in the thermal decomposition of HP-β-CD, which is around 350 °C, but it decreased to ∼300 °C at a Px:HP-β-CD molar ratio of 1:1. Similar thermal behavior was observed for flibanserin/HP-β-CD inclusion complexes^52^ and azomethine β-CD inclusion complexes,^53^ where inclusion complexation decreased the thermal stability of CDs.

**Figure 6:**
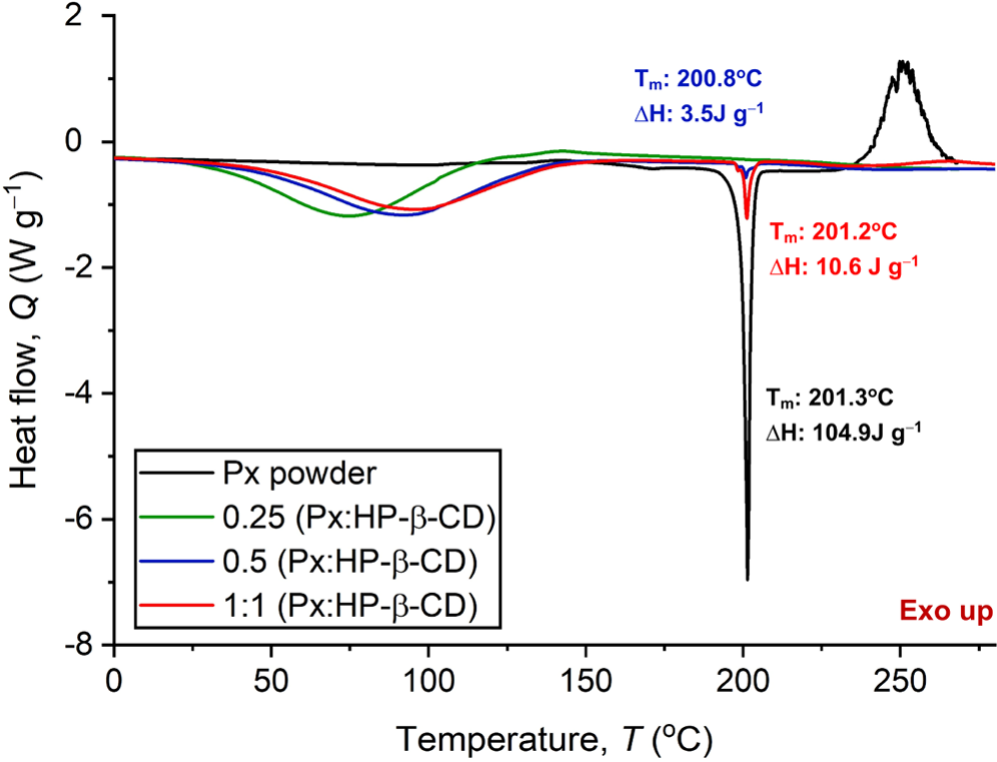
DSC curves of the Px powder and HP-β-CD/Px fibers of various molar ratios.

Figure 6 shows the DSC curves of the Px powder and HP-β-CD/Px fibers for the temperature range of 0–270 °C. A sharp endothermic peak of Px appeared at 201.3 °C owing to its melting. The DSC curve of the Px:HP-β-CD (0.25:1) also showed a broad peak due to the evaporation of water molecules while no melting peak of Px could be detected. Further increasing the Px content to 0.5 revealed a small melting peak due to uncomplexed Px. With increasing the Px content to 1:1, the intensity of the melting peak of uncomplexed Px slightly increased, demonstrating that most of the Px could be hosted in the HP-β-CD cavity.

**Figure 7:**
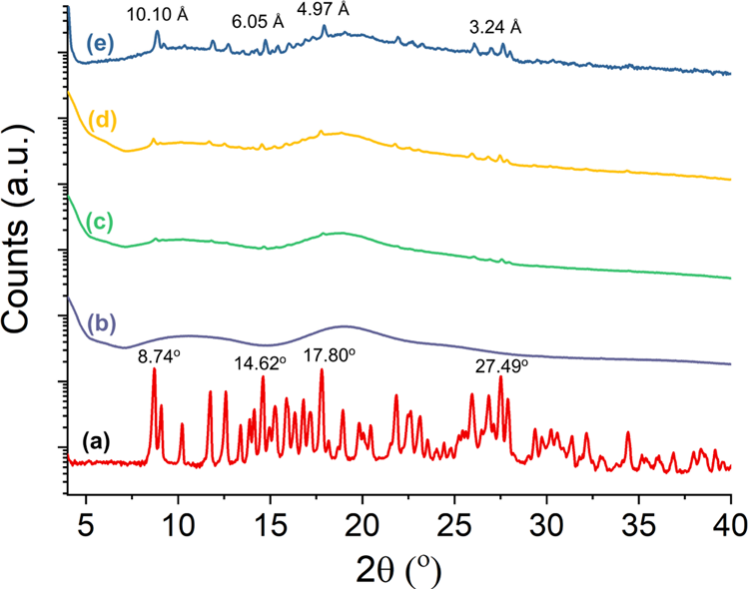
Wide-angle XRD patterns of the (a) Px, (b) HP-β-CD fibers, and Px/HP-β-CD fibers of various molar ratios (c) 0.25:1, (d) 0.5:1, and (e) 1: 1, respectively.

The formation of inclusion complexation between HP-β-CD and Px was also confirmed by wide-angle XRD analysis. Figure 7 shows the XRD patterns of the Px, HP-β-CD, and HP-β-CD/Px nanofibers produced at various molar ratios. The XRD pattern of Px might depict a cubic crystal polymorph, as previously reported.^54^ The XRD pattern of HP-β-CD fibers shows broad, amorphous peaks centered at 10.5 and 18.8°, in line with the literature reports.^55^ Unlike HP-β-CD, Px is highly crystalline and shows many sharp peaks between 8 and 30°. The most pronounced ones appeared at 8.74, 14.62, 17.80, and 27.6°, with the respective *d*-spacing values of 10.10, 6.05, 4.97, and 3.24 Å. These dominant peaks significantly disappeared for the IC fibers with a lower Px content, demonstrating that Px could form ICs with HP-β-CD. They become apparent in the fibers containing a higher Px content due to uncomplexed Px molecules. However, most Px could form inclusion complexes with HP-β-CD, as demonstrated by both DSC and XRD analyses. Molecular dynamics simulations on the inclusion complexation of Px with HP-β-CD were previously reported,^56^ where the authors found that the benzothiazine of Px entered the cavity from the primary side of HP-β-CD molecules. Px solubility, FTIR spectra, and XRD patterns confirm the inclusion complexation between the HP-β-CD and Px. However, at very high Px contents, the presence of the uncomplexed Px could be seen in the XRD pattern. At mixing times (<1 h), the solution was not homogeneous with clearly seen particles and precipitation. While increasing the mixing time to 1 day, no precipitation of Px was observed owing to the solubilization of Px by IC formation.

**Figure 8:**
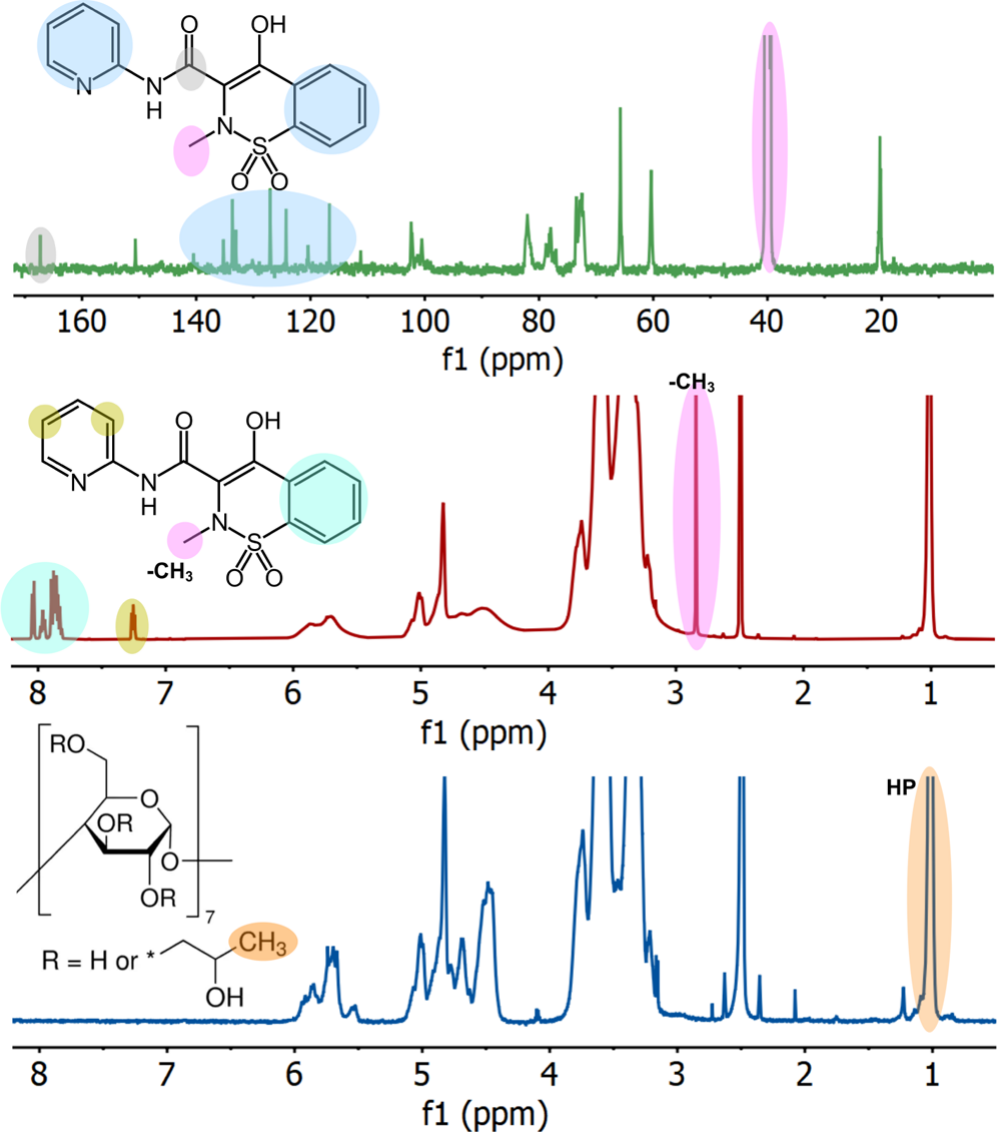
^1^H and ^13^C NMR spectra of the HP-β-CD fibers and HP-β-CD/Px fibers (1:0.5) in *d*_6_-DMSO.

Px preservation in the fibers was explored after six months of incubation at RT through NMR analysis. Figure 8 shows the ^1^H and ^13^C NMR spectra of HP-β-CD and Px:HP-β-CD fibers (0.5:1) produced. The methyl (−CH_3_) protons peak of the hydroxypropyl group (HP-β-CD) appeared at 1 ppm, while the backbone proton peaks of HP-β-CD appeared between 3 and 6 ppm. On the other hand, HP-β-CD fibers containing Px showed a sharp peak at around 2.84 ppm of the methyl proton of Px, while aromatic protons of Px appeared at 7.83–8.05. Aromatic protons in the pyridine ring of Px were visible between 7.24 and 7.27 ppm. ^13^C NMR of the Px/HP-β-CD nanofibers showed a peak at 40.5 ppm due to CH_3_ of HP-β-CD and Px, and the peaks between 116.7 and 135.3 ppm can be denoted as the aromatic carbon atoms of Px, while the carbonyl carbon of Px appeared at 167.3 ppm. The appearance of Px peaks in the HP-β-CD fibers after 6 months of incubation clearly shows the preservation of Px within the fibers. This is particularly important for showing the long shelf-life of the HP-β-CD nanofiber-based drug delivery system. The loading amount of Px was calculated from NMR data using methyl (−CH_3_) protons of Px and HP-β-CD. The ^1^H NMR findings revealed that the nanofibers with 0.5:1 and 1:1 molar ratios have an approximate Px loading capacity of 10.2 and 17.3 wt %, respectively. The respective encapsulation efficiency was calculated to be 93 and 99%.

**Figure 9:**
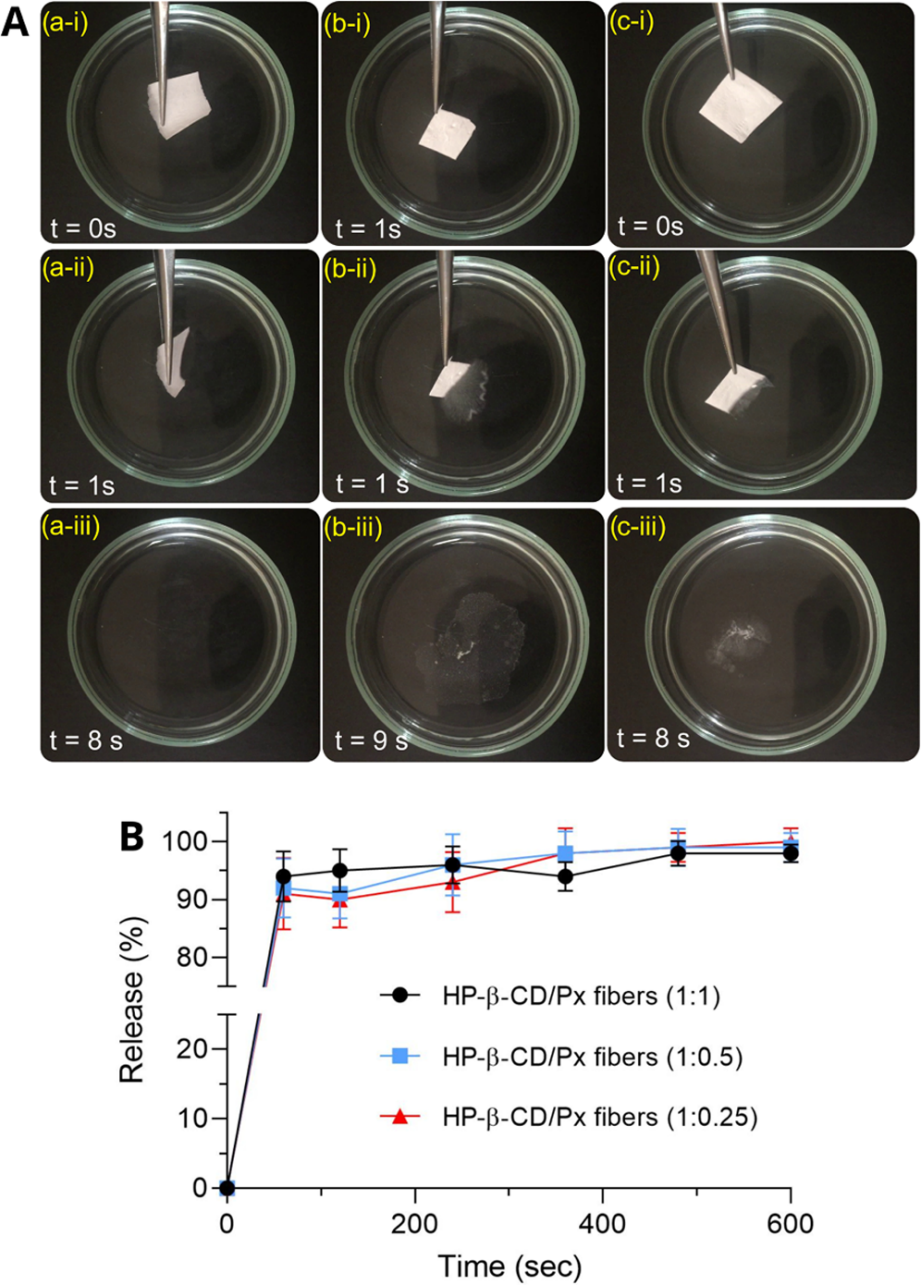
(A) The dissolution of the HP-β-CD/Px nanofibers prepared at various molar ratios (a) HP-β-CD control; (b) 0.25: 1 (Px: HP-β-CD); and (c) 0.5: 1 (Px: HP-β-CD) in artificial saliva. The photos were captured from Supporting Information Video 1. (B) Time-dependent *in vitro* release of Px from the HP-β-CD/Px nanofibers of different compositions in PBS (pH: 7.4).

The dissolution behavior of the nanofibers was explored using artificial saliva (Figure 9A). Because of its polymer-free and hydrophilic nature, HP-β-CD/drug IC nanofibers having different Px contents instantly dissolved in artificial saliva on contact (Supporting Information, Videos 1 and 2). The dissolution of each fiber mat occurred within 1 s. These results align with the previous reports on polymer-free CD-based nanofibers, where the fibers, because of the hydrophilic nature of CD, instantly dissolved in aqueous media.^41^ The *in vitro* release of the Px from the nanofibers was explored in PBS at 37 °C using a UV–vis spectrophotometer. Figure 9B shows the time-dependent Px release from the HP-β-CD/Px nanofibers (1:1, 1:0.5, and 1:0.25). A burst release was observed for all nanofibers, and over 90% of Px could be released in a minute: 94 ± 4.3, 92 ± 5.1, and 91 ± 6.2% of Px could be released in 60 s for the nanofibers with 1:1, 1:0.5, and 1:0.25 molar ratios, followed by a plateau region for all nanofibers. The burst release of Px could be attributed to the rapid dissolution of the nanofibers. After 10 min, the Px release percentages for both nanofibers were 98 ± 1.9, 99 ± 2.5, and 100 ± 2.3, respectively, demonstrating that all of the Px could be released. The *in vitro* release results clearly demonstrate that the nanofibers drastically increase the release profile of Px in aqueous media owing to the formation of IC between the HP-β-CD and Px. This rapid release profile could also be attributed to the very high water solubility of HP-β-CD (∼2000 mg mL^–1^).

**Figure 10:**
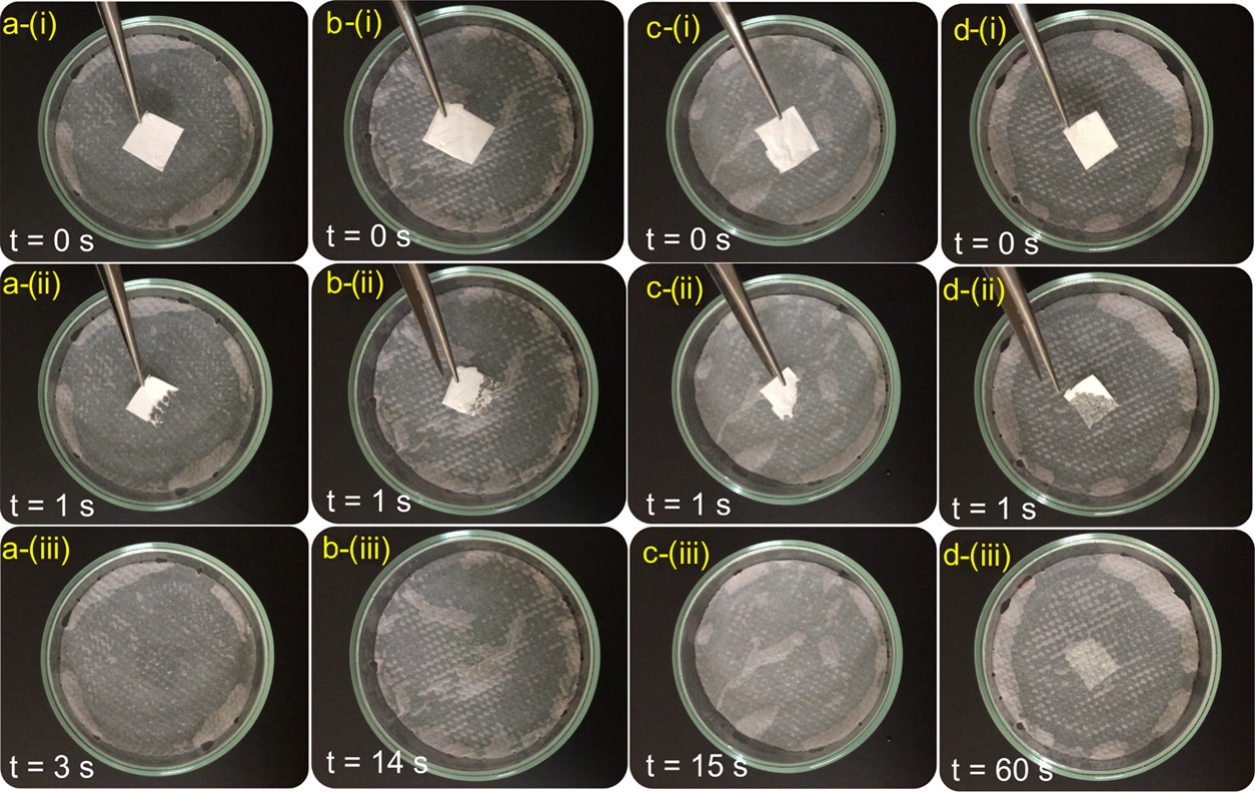
Dissolution of the HP-β-CD (a) and Px/HP-β-CD nanofibers prepared at various molar ratios ((b) 0.1:1, (c) 0.25:1 and (d) 0.5:1 with respect to HP-β-CD) on tissues wetted with artificial saliva. The photos were captured from Supporting Information Video 2.

To mimic the sublingual delivery of drugs, the experiments were performed on tissues wetted with artificial saliva, and dissolutions of the fibers were recorded by a video camera (Figure 10). The sample with low Px loadings disappeared instantly because of the polymer-free hydrophilic nature of CD molecules. Increasing the Px content decreased the dissolution time, but the fibers dissolved in less than a minute, demonstrating their suitability for sublingual drug delivery. Overall, the use of polymer-free CD nanofibers for oral delivery of the embedded drugs offers great potential for rapid oral delivery of drugs. In this regard, various systems have been reported for rapid oral delivery of drugs, such as antiemetic drugs ondansetron,^57^ hydrocortisone,^58^ and ibuprofen.^59^ Because of hydrophilic and uncross-linked structure of CD nanofibers, the drug release occurs very fast. However, the drug release could be slowed down by the use of a polymer carrier, such as pullulan.^60^

## 4 Conclusions

Waterborne electrospinning of polymer-free HP-β-CD/Px IC nanofibers without using a carrier polymer was reported. Bead-free fibers with a mean diameter range of 170–500 nm were produced; increasing the Px content led to a decrease in the fiber diameter. Despite the polymer-free nature of the fibers, the mats could be folded many times without any crack development. FTIR and TGA revealed the presence of Px within the fibers, while XRD analysis revealed the formation of inclusion complexation between the HP-β-CD and Px over the disappearance of crystalline peaks of Px. DSC also revealed the disappearance of the melting peak of Px as a result of IC formation. NMR analysis revealed Px preservation within the fibers after six months of storage, demonstrating that inclusion complexation could improve the stability of piroxicam within the CD cavity. Because of the hydrophilic and cross-link-free structure, HP-β-CD/Px fibers instantly dissolved in artificial saliva, while the dissolution could be slowed down with the use of tissues wetted with artificial saliva (*i*.*e*., to mimic oral delivery). Px molecules were released to the medium as HP-β-CD/Px ICs. Overall, this is the first time that the production of Px-loaded polymer-free electrospun fibers, and the concept relies on waterborne electrospinning without any organic solvents. Since Px could be solubilized by inclusion complexation with CD molecules, high drug loading capacity could be reached without using organic solvents, and the resultant fibers are instantly dissolved in artificial saliva, demonstrating the great potential of the concept for sublingual drug delivery. Overall, the novelty of this paper is as follows:

(i) First-time production of polymer-free electrospinning of Px-loaded fibers.
(ii) The concept relies on waterborne electrospinning without using any organic solvents for drug solubilization and fiber production.
(iii) Px could be solubilized by CD molecules in water so that the Px could be loaded at high proportions in the fibers.
(iv) The fiber mat rapidly dissolved in artificial saliva, releasing Px, which is desired for the sublingual delivery of drugs.

## Supporting Information Available

Scanning electron micrographs of Px powder and control HP-β-CD nanofibers; a photograph of aqueous solutions of HP-β-CD/Px (PDF)
Dissolution of the HP-β-CD/Px nanofibers in artificial saliva (AVI)
Dissolution of the HP-β-CD and HP-β-CD/Px nanofibers on tissues wetted with artificial saliva (AVI)

## Supplementary Material

ao2c03987_si_001.pdf

ao2c03987_si_002.avi

ao2c03987_si_003.avi
