# Supplementary material for: Rapid Sublingual Delivery of Piroxicam from Electrospun Cyclodextrin Inclusion Complex Nanofibers
Source: ACS Omega. 2022 Sep 19;7(39):35083–91. doi: 10.1021/acsomega.2c03987 (PMC9535703; doi:10.1021/acsomega.2c03987)
Supplement: Supplementary file 1 — ao2c03987_si_001.pdf [file ao2c03987_si_001.pdf]

Supporting Information

for

Rapid Sublingual Delivery of Piroxicam from Electrospun Cyclodextrin Inclusion-Complex Nanofibers

Fuat Topuz*

Department of Chemistry, Faculty of Science and Letters, Istanbul Technical University, Sariyer 34467, Istanbul (Turkey)

*Corresponding author: Dr. F. Topuz (Email: topuzf@itu.edu.tr & fuat.topuz@rwth-aachen.de)

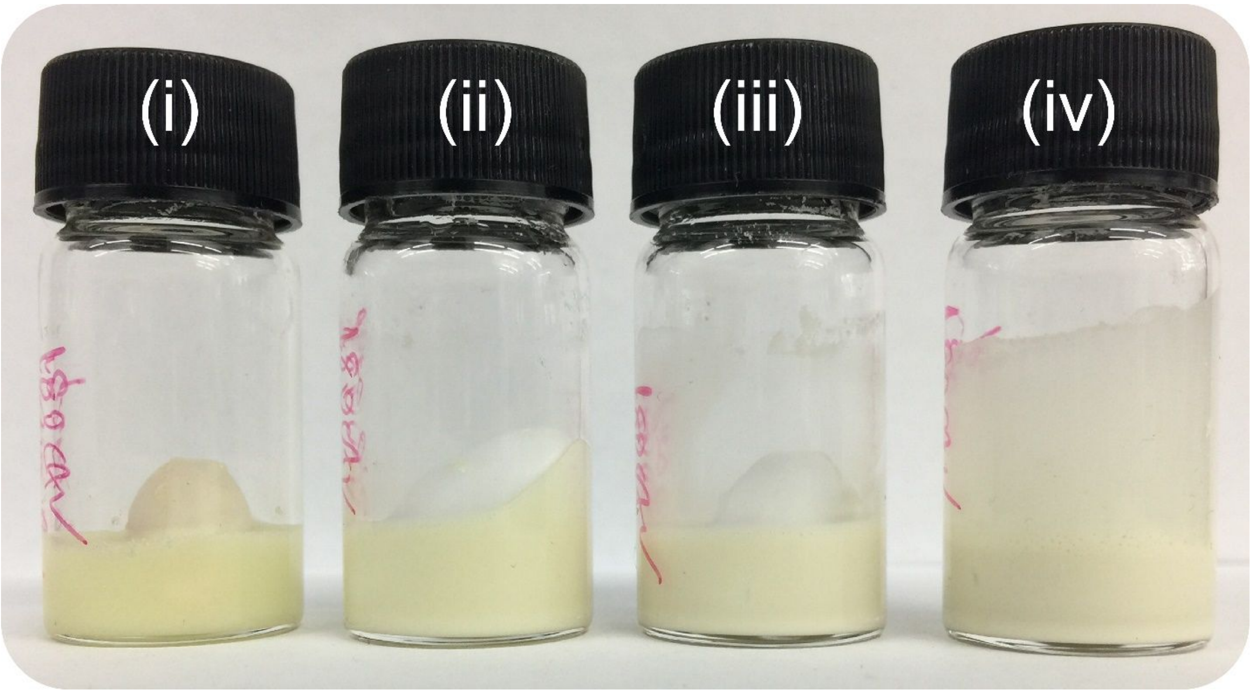

Figure S1. A photograph of aqueous solutions of HP-β-CD/Px at various molar ratios ((i) 0.1:1, (ii) 0.25:1, 0.5:1 and 1:1).

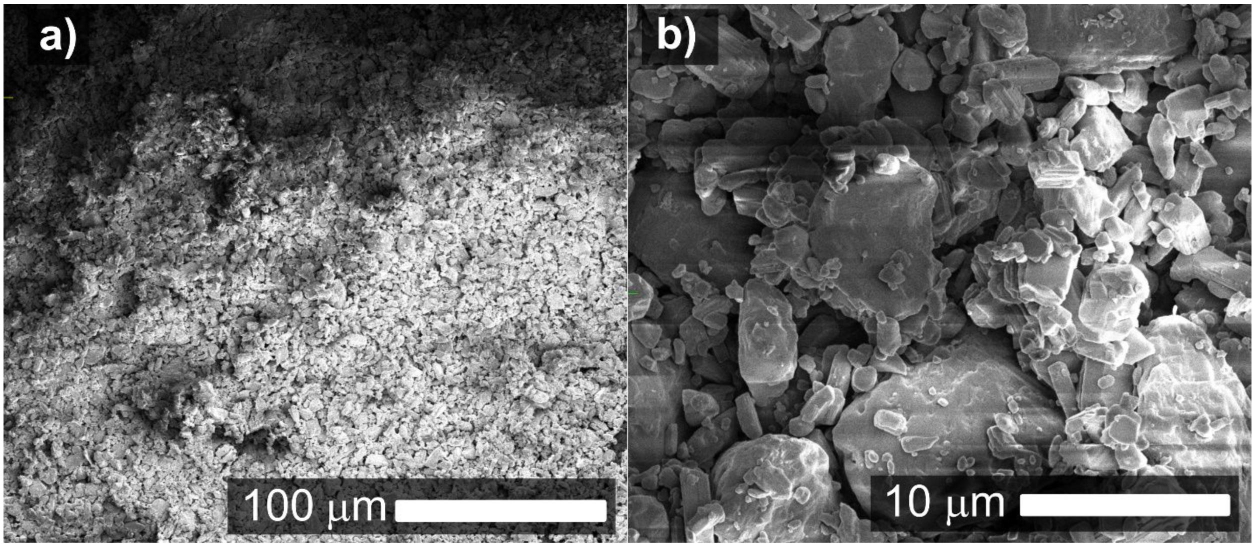

Figure S2. Scanning electron micrographs of piroxicam (Px) powder at different magnifications (a, b)

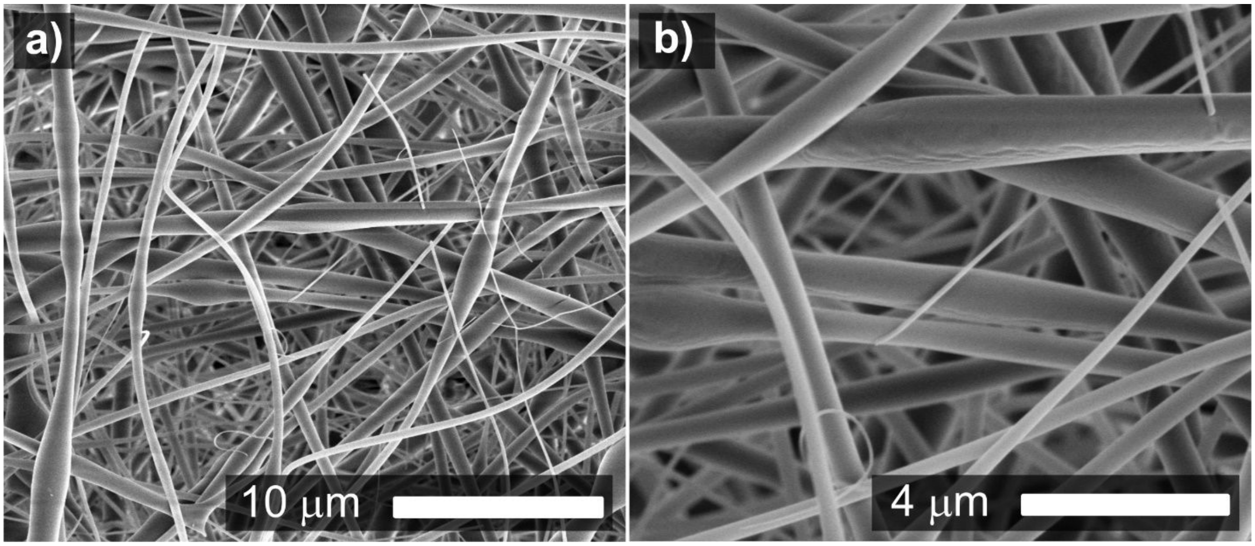

Figure S3. (a, b) Scanning electron micrographs of HP-*β*-CD fibers at different magnifications produced at 180% (w/v) in water. The applied voltage : 15 kV, the tip-to-collector distance : 15 cm, and the flow rate : 0.5 mL h^{-1}.
